# Evaluation of multi-antigen serological screening for active tuberculosis among people living with HIV

**DOI:** 10.1371/journal.pone.0234130

**Published:** 2020-06-04

**Authors:** Devan Jaganath, Jayant Rajan, Christina Yoon, Resmi Ravindran, Alfred Andama, Lucy Asege, Sandra Z. Mwebe, Jane Katende, Martha Nakaye, Fred C. Semitala, Imran H. Khan, Adithya Cattamanchi

**Affiliations:** 1 Department of Pediatrics, Division of Pediatric Infectious Diseases, University of California, San Francisco, CA, United States of America; 2 Department of Medicine, Division of Pulmonary and Critical Care Medicine, University of California, San Francisco, CA, United States of America; 3 Department of Medicine, Center for Tuberculosis, University of California, San Francisco, CA, United States of America; 4 Department of Medicine, Division of Experimental Medicine, University of California, San Francisco, CA, United States of America; 5 Department of Pathology and Laboratory Medicine, University of California, Davis, CA, United States of America; 6 Infectious Disease Research Collaboration, Kampala, Uganda; 7 Department of Medicine, Makerere University College of Health Sciences, Kampala, Uganda; 8 Makerere University Joint AIDS Program (MJAP), Kampala, Uganda; 9 Department of Medicine, Center for Vulnerable Populations, University of California, San Francisco, CA, United States of America; 10 Curry International Tuberculosis Center, University of California, San Francisco, CA, United States of America; Rutgers Biomedical and Health Sciences, UNITED STATES

## Abstract

Better triage tests for screening tuberculosis (TB) disease are needed for people living with HIV (PLHIV). We performed the first evaluation of a previously-validated 8-antigen serological panel to screen PLHIV for pulmonary TB in Kampala, Uganda. We selected a random 1:1 sample with and without TB (defined by sputum culture) from a cohort of PLHIV initiating antiretroviral therapy. We used a multiplex microbead immunoassay and an ensemble machine learning classifier to determine the area under the receiver operating characteristic curve (AUC) for Ag85A, Ag85B, Ag85C, Rv0934-P38, Rv3881, Rv3841-BfrB, Rv3873, and Rv2878c. We then assessed the performance with the addition of four TB-specific antigens ESAT-6, CFP-10, Rv1980-MPT64, and Rv2031-HSPX, and every antigen combination. Of 262 participants (median CD4 cell-count 152 cells/μL [IQR 65–279]), 138 (53%) had culture-confirmed TB. The 8-antigen panel had an AUC of 0.53 (95% CI 0.40–0.66), and the additional 4 antigens did not improve performance (AUC 0.51, 95% CI 0.39–0.64). When sensitivity was restricted to ≥90% for the 8- and 12-antigen panel, specificity was 2.2% (95% CI 0–17.7%) and 8.1% (95% CI 0–23.9%), respectively. A three-antigen combination (Rv0934-P38, Ag85A, and Rv2031-HSPX) outperformed both panels, with an AUC of 0.60 (95% CI 0.48–0.73), 90% sensitivity (95% CI 78.2–96.7%) and 29.7% specificity (95% CI 15.9–47%). The multi-antigen panels did not achieve the target accuracy for a TB triage test among PLHIV. We identified a new combination that improved performance for TB screening in an HIV-positive sample compared to an existing serological panel in Uganda, and suggests an approach to identify novel antigen combinations specifically for screening TB in PLHIV.

## Introduction

Tuberculosis (TB) is the leading cause of death among HIV-infected individuals worldwide [1] and the World Health Organization (WHO) recommends routine TB screening for all people living with HIV (PLHIV) [2]. Optimal triage tests for TB should be used to screen at risk-groups [3], but current approaches have insufficient diagnostic accuracy among PLHIV. The WHO four-part symptom screen lacks specificity [4], especially if the individual is severely immunosuppressed [5–7]. While gene expression signatures have shown some promise [8], translating them to a point-of-care diagnostic is a challenge [9]. There is an urgent need for an affordable, non-sputum, biomarker-based test that achieves the minimum accuracy thresholds (90% sensitivity and 70% specificity) recommended by the WHO for a TB triage test [10].

Antibodies remain popular candidate TB biomarkers [9], given the ease of obtaining a blood sample and potential for a point-of-care assay [11], However, due to high variability in sensitivity and specificity [11], the WHO recommends against using current commercial serologic tests for TB screening or diagnosis [12]. Yet, these assays have primarily examined the antibody response to individual antigens [9], and recent studies have found greater success with multiple antigen panels [13–15].

A systematic-review on TB biomarkers highlighted two promising studies in Uganda and Pakistan that used a rapid, high throughput, and affordable multiplex microbead immunoassay [9, 13, 15]. In both settings, antibody responses to 28 TB antigens were simultaneously measured in hospitalized HIV negative adults with pulmonary symptoms and being evaluated for TB. The individual antigens were prioritized by ability to discriminate TB status, and machine learning methods were used to determine the best combination that could serve as a triage test. In Kampala, Uganda, Shete et al., found an 8-antigen panel with 90.6% sensitivity and 88.6% specificity [13], and in Lahore, Pakistan, Khaliq et al. reported an 11-antigen panel with a sensitivity of 91% and specificity of 96% [15]. The Uganda 8-antigen panel shared four antigens with the 11-antigen panel, and three of the remaining four were among the first or second tier priority antigens in Pakistan [16]. Both achieved the target profile for a triage test, but have not been assessed in the context of TB screening among high-risk groups such as PLHIV. Given that HIV positive individuals are immunosuppressed and TB screening for PLHIV is often done in the outpatient setting, it is important to examine the performance of multi-antigen serological panels in this context.

Among adults initiating anti-retroviral therapy (ART) at two HIV clinics in Uganda, our objectives were to 1) Evaluate the accuracy of the 8-antigen panel; 2) Assess any improved performance with four additional TB antigens ESAT-6, CFP-10, Rv1980-MPT64, and Rv2031-HSPX; and 3) Determine the best performing combination of the 12 antigens to screen for TB disease.

## Materials and methods

### Population

We conducted a nested case-control study within a prospective cohort of adult PLHIV initiating anti-retroviral therapy (ART) in Kampala, Uganda [17]. Participants were consecutively enrolled from two HIV clinics at Mulago Hospital National Referral Hospital from July 2013 to December 2015. Individuals were included if they were 18 years and older and were eligible for ART at the time with CD4 cell count ≤ 350 cells/μL in the prior 3 months. They were excluded if they already had a diagnosis of active TB disease or had taken ART or any medication with anti-mycobacterial activity (i.e. TB treatment, isoniazid preventative therapy, or fluoroquinolones) in the last 3 days. From this cohort (N = 1,177), we selected a 1:1 random sample of 292 participants (based on specimen availability, complete data, and cost) with and without culture-confirmed TB diagnosed at the time of enrollment. All participants completed an informed written consent, and the study was approved by the University of California, San Francisco and Makerere University School of Medicine Institutional Review Boards, as well as the Uganda National Council for Science and Technology. The study conformed to the Standards for the Reporting of Diagnostic Accuracy Studies (STARD) initiative [18], and our analyses further followed the Transparent Reporting of a multivariable prediction model for Individual Prognosis or Diagnosis (TRIPOD) guidelines [19].

### Procedures

Trained staff collected demographic and clinical data, and performed the WHO four-symptom TB screen of current cough, fever, night sweats and weight loss [4]. All participants submitted two spot expectorated sputum samples at the time of enrollment for Xpert MTB/RIF (Cepheid, Sunnyvale, CA, USA) testing and liquid culture (BACTEC 960 Mycobacterial Growth in Tube [MGIT) system, Becton Dickenson, Franklin Lakes, NJ, USA). Solid culture with Löwenstein-Jensen media was performed in addition to liquid culture from June 10, 2014 to December 1, 2015. To confirm *M*. *tuberculosis*, positive cultures underwent acid-fast bacilli (AFB) smear microscopy and molecular speciation testing (Capilia TB, TAUNS, Japan, or MPT64 assay, Standard Diagnostics, South Korea), per standard protocol by the Mycobacteriology laboratory. Blood samples from all participants were collected in EDTA tubes, centrifuged and plasma stored in -80°C freezers, and maintained with standard quality control protocols, temperature monitoring and continuous backup power. There were no prior freeze-thaw cycles for the samples tested.

### Multiplex microbead immunoassay

#### Microbead coating with *M*. *tuberculosis* antigens

In brief, the antigens were expressed in *Escherichia coli* as recombinant histidine-tagged products and purified to near-homogeneity for bead coupling as previously detailed [20]. We assessed the 8 antigens found to be useful for TB screening in Uganda (Ag85A, Ag85B, Ag85C, Rv0934-P38, Rv3881, Rv3841-BfrB, Rv3873, and Rv2878c) [13]. In addition, we included 4 antigens (ESAT-6, CFP-10, Rv1980-MPT64, and Rv2031-HSPX) that were in the first or second priority tier in Pakistan and are well-known targets for TB diagnosis [21–24].

Carboxylated microbeads were purchased from Luminex Corp. (Austin, TX). Various antigen preparations were covalently conjugated to the microbeads according to the manufacturer's instructions. Briefly, bead stock was resuspended by vortexing and treatment in a sonicator bath (15 to 30 s) (Branson 1510; Danbury, CT). An aliquot of 2.5 × 10^6^ beads was removed and centrifuged at 21,000 × g for 2 min. Beads were resuspended in 80 μl of activation buffer (100 mM monobasic sodium phosphate; pH 6.3) by vortexing and sonication (15 to 30 s). To activate the beads for cross-linking to proteins, 10 μl of 50-mg/ml sulfo-N-hydroxysulfosuccinamide (Pierce, Rockford, IL) was added, and beads were mixed by vortexing. Then 10 μl of 50-mg/ml 1-ethyl-3-[3-dimethylaminopropyl]carbodiimide (EDC; Pierce, Rockford, IL) was added, and beads were mixed again by vortexing. All incubations of beads were performed in the dark. The bead mixture was shaken on a rotary shaker at room temperature for 20 min and centrifuged at 21,000 × g for 2 min. Beads were washed twice with 250 μl of wash buffer (phosphate-buffered saline (PBS), pH 7.4). To coat them with antigens, pelleted beads were resuspended in the relevant antigen preparation diluted in PBS (pH 7.4) buffer. For coupling, mixtures of activated beads and proteins were incubated by shaking on a rocker for 2 h at room temperature. After coating with proteins, beads were washed twice with 250 μl of wash buffer (PBS, pH 7.4), resuspended in 250 μl of blocking buffer (1% BSA; 0.1% Tween 20 in PBS, pH 7.4; 0.05% sodium azide), and shaken on a rocker at room temperature for 30 min. After blocking, beads were resuspended in 1 ml of blocking buffer and stored at 4°C.

The optimal concentration for each antigen was determined by coupling different microbead sets (2.5 × 10^6^ beads/coupling) with a range of protein concentrations between 4 and 100 μg/ml for each antigen. Coated microbeads for each antigen were tested with sera from TB patients which are positive for antibodies to the relevant antigen. Antigen concentration that displayed the strongest specific signal for each antigen against the positive TB patient sera and lowest background (against healthy sera) were selected. The optimized protein concentration for each antigen was as follows: 6.25 ug/ml of Ag85A, 25 μg/ml of Ag85B, 100 ug/ml of Ag85C, 4 μg/ml of Rv0934-P38, 12.5 μg/ml of Rv3881, 100 μg/ml of Rv3841-BfrB, 25 μg/ml of Rv3873, 25 μg/ml of Rv2878c, 6.25 μg/ml of ESAT-6, 12.5 μg/ml CFP-10, 25 μg/ml of Rv1980-MPT64, and 25 μg/ml of Rv2031-HSPX. Bead sets were also coated with bovine serum albumin (BSA, 100 μg/ml) as a negative control protein (Pierce, Rockford, IL).

#### Multiplex immunoassay

We used a multiplex microbead immunoassay based on the xMAP platform (Luminex Corp, Austin, TX) to evaluate the IgG response to *M*. *tuberculosis* antigens as described in prior studies [13, 15, 16, 25]. Multiplex assays were performed and data (median fluorescence intensity [MFI]) were collected as previously described [26, 27]. In brief, a mixture of microbead sets, one for each of the coated antigens described above, were incubated with the participants’ plasma specimens, which were diluted 1:200 in Prionex (bio-WORLD, Dublin, OH) for 2 hours at room temperature. After incubation, liquid was drained from the bottom of the plate in a vacuum manifold designed to hold 96-well plates (Millipore Corporation, Bedford, MA). The beads were washed two times by adding 100 μl of wash buffer per well and draining under vacuum. For detection of human IgG, phycoerythrin conjugated anti-human IgG was used (Jackson ImmunoResearch, Pennsylvania) at a 1:500 dilution in PBS-tween, and 50 μl was added per well. Beads were mixed as before and incubated at room temperature for 30 min. Following incubation with the secondary antibody, beads were washed two times with wash buffer, resuspended in 100 μl of wash buffer per well, and analyzed in the Luminex-100 instrument.

The frozen samples were thawed only once, two technical replicates were performed, and the MFI measured in August 2016. BSA was used as a negative control to determine background fluorescence, and was subtracted from the MFI to provide a measure of reactivity. We excluded samples if the negative control background fluorescence was high (MFI > 200), or if participants without TB had a high antibody response (> 3 standard deviations (SD) from the mean).

### Reference standard

Participants with confirmed pulmonary TB were defined as having at least one sputum culture with *M*. *tuberculosis* from two samples. Individuals with two negative cultures were defined as not having TB. We did not include samples from participants who were culture-negative but started anti-TB treatment empirically or who had follow up sputum specimens that were positive for TB. The staff performing the index testing were blinded to the TB status of the participant.

### Statistical methods

We summarized demographic and clinical characteristics of the sample with descriptive statistics. We log transformed antibody titers as measured by MFI, and responses to individual antigens by TB status were visualized using boxplots and compared by permutation testing, with significance defined as p-value < 0.05. We randomly partitioned the full dataset into a training set (2/3) and an independent test set (1/3). To assess the performance of the 8-antigen set in Uganda, we used the same approach as those authors by creating an ensemble machine learning classifier with the R package Super Learner on our training data [28], using the same library of modeling approaches (logistic regression, Bayes’ generalized linear models (GLM), lasso, and random forests), and 10-fold cross validation [13]. We then used the trained model to generate predictions on the test dataset, and calculated the area under the receiver operating characteristic curve (AUC), and specificity when sensitivity was set at ≥90%. We created 500-bootstrapped test dataset samples to generate 95% confidence intervals (CIs). We applied the same approach with the expanded set of 12 TB-specific antigens, training the data using an ensemble of lasso, random forests, Bayesian generalized linear models (GLM), generalized additive models (GAM), and neural networks. These methods were selected by evaluating their individual performance based on mean squared error (MSE) on the training data, and they had the lowest and similar MSE (range 0.26–0.29) compared to other algorithms (*k*-nearest neighbors, GLM, naïve Bayes classifier, support vector machines, and forward stepwise regression, MSE range 0.36–1.97).

Finally, we also examined the performance of all 1–12 antigen linear combinations (n = 8,191) of the 12 antigens in the training dataset, using a previously described approach to identify a novel gene expression signature in the same cohort [29]. We computed ROC curves for each combination across 500 bootstrapped training datasets and identified the combination that had the highest mean AUC. We then tested the performance of this combination on the test dataset and created an ROC curve to determine the cut-off that maximized specificity when sensitivity was ≥90%. We calculated the sensitivity and specificity at that cut-off with exact binomial 95% CIs, and compared the specificity to the WHO four-symptom TB screen using McNemar’s test (significance defined as p-value < 0.05). All analyses were done using R version 3.5.1 (www.r-project.org/), R Studio version 1.1.456, and the *SuperLearner* (version 2.0–24) [30] and *ROCR* (version 1.0–7) [31] packages. The data is available in the supporting information (S1 File).

## Results

### Sample characteristics

**Fig 1:**
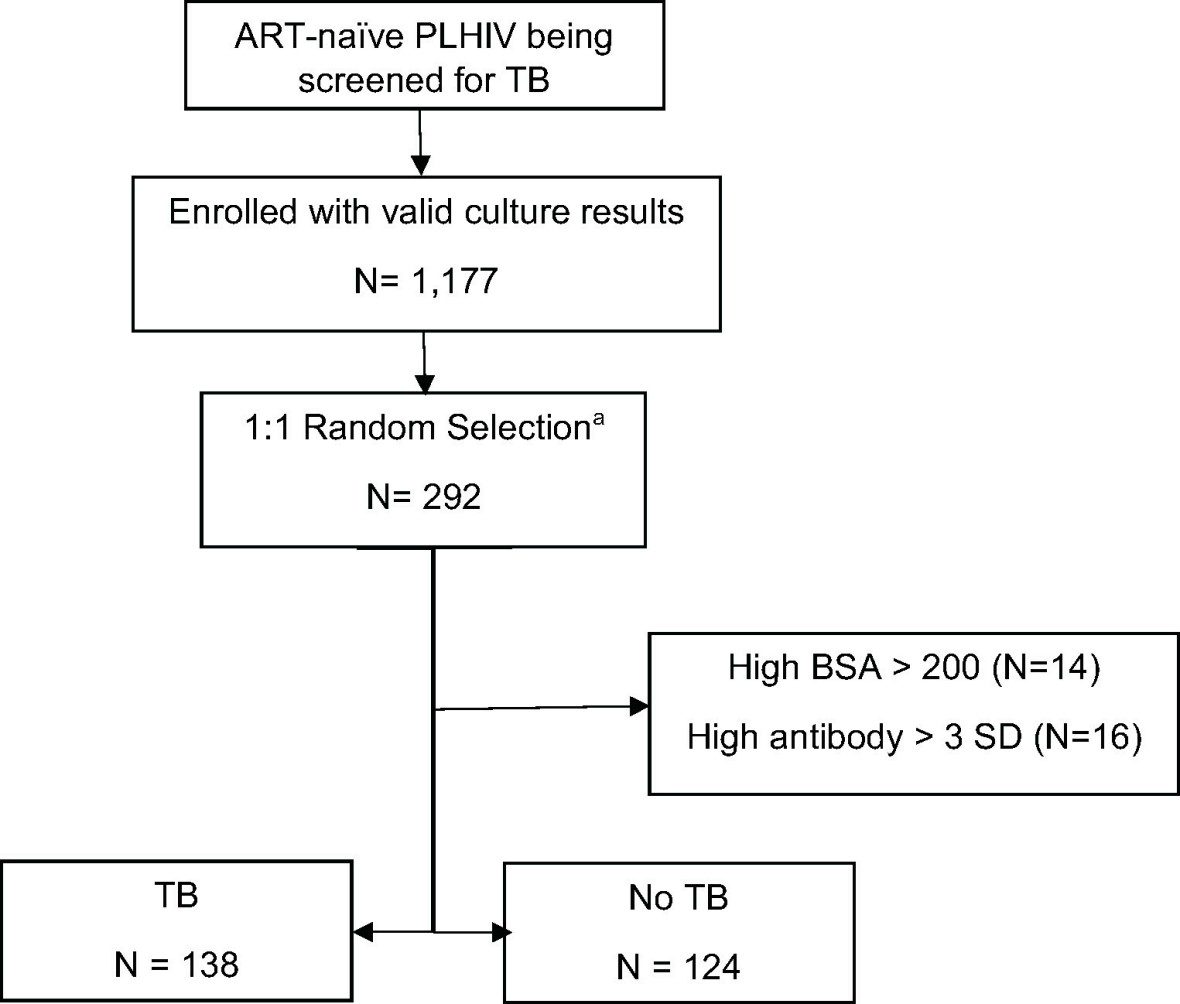
Flowchart of participants. a. 292 selected based on specimen availability, complete data, and cost ART: antiretroviral therapy; BSA: Bovine Serum Albumin; PLHIV: People living with HIV; SD: Standard Deviation; TB: tuberculosis.

We selected 292 participants from the cohort, of whom 146 (50%) had pulmonary TB (Fig 1). Fourteen participants (8 from TB group, 6 from non-TB group) had a negative control BSA MFI >200 and were removed from the analysis. An additional 16 participants without TB had a high antibody level > 3 SD and were excluded, resulting in a final sample of 138 with TB and 124 without TB. Of those without TB, Xpert MTB/RIF testing agreed with culture in 99% of participants (123/124), with one participant having a low Xpert MTB/RIF semi-quantitative result, but two negative sputum smears and cultures. Of those with TB, two-thirds (67%, 93/138) were smear negative.

The median age was 34 years (IQR 27–41) and 52% were male (95% CI 46–58). The median CD4 cell-count was 152 cells/μL (IQR 65–279) and 70 (27%, 95% CI 22–32) were underweight (BMI <18.5). There were 239 (91%) participants that met at least one criteria for the WHO TB symptom screen, although 104 (40%) did not have a cough at the time of evaluation.

### Individual antibody responses

**Fig 2:**
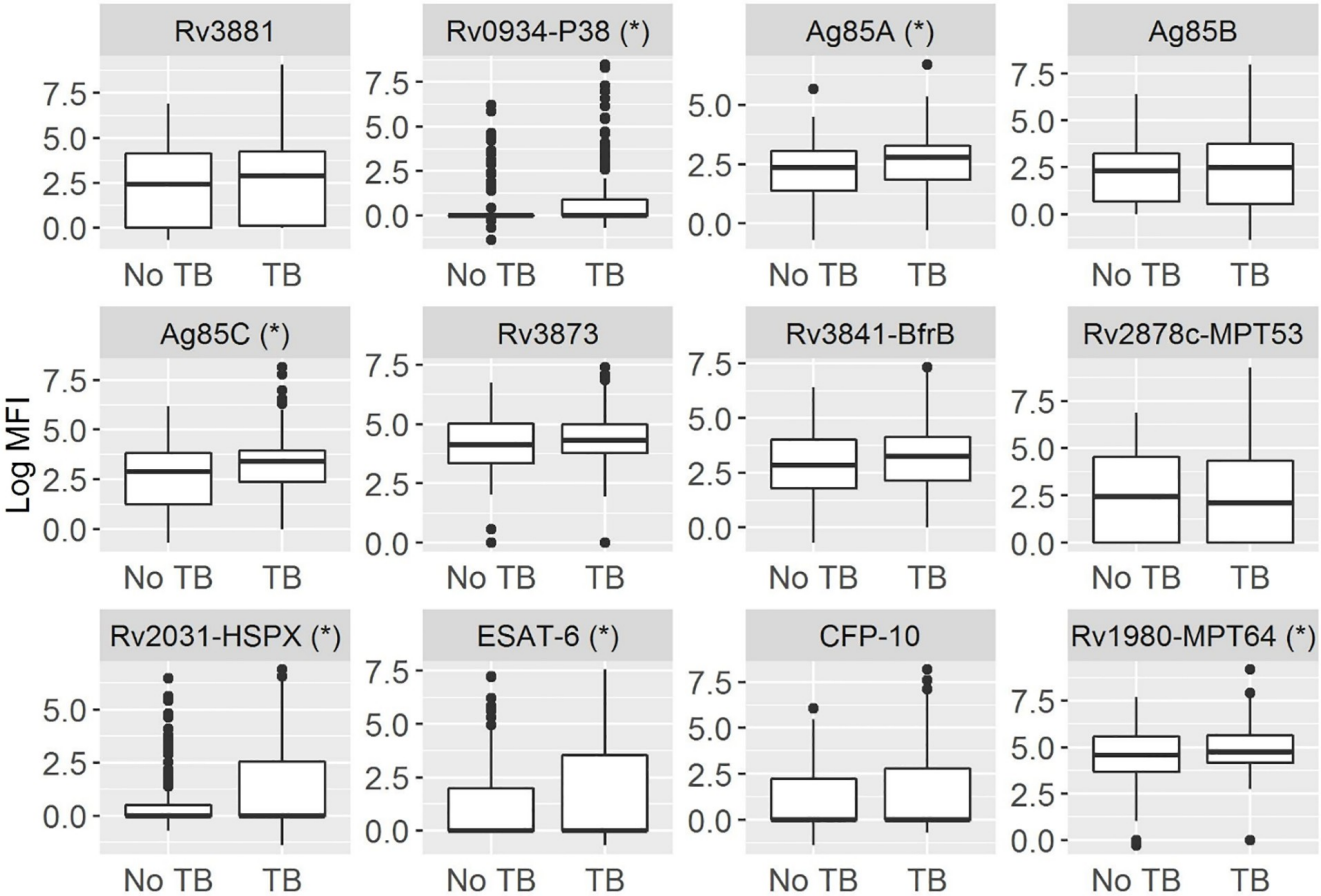
Boxplots of antibody responses to TB antigens by TB status. With a multiplex microbead immunoassay, we compared the log median fluorescence intensity (MFI) among HIV positive adults with and without TB. A star next to the antigen indicates a significant difference by permutation testing (p-value < 0.05).

We compared the antibody responses to the 12 individual antigens by TB status (Fig 2 and S1 Table). Several antigens had a median log MFI of 0 (Rv0934-P38, Rv2031-HSPX, ESAT-6, and CFP-10). We found small but significant elevations in antibody responses in TB vs. non-TB participants for antigens Rv0934-P38, Ag85A, Ag85C, Rv2031-HSPX, ESAT-6 and Rv1980-MPT64 (S1 Table).

### Diagnostic accuracy of 8-antigen and 12-antigen panels

**Fig 3:**
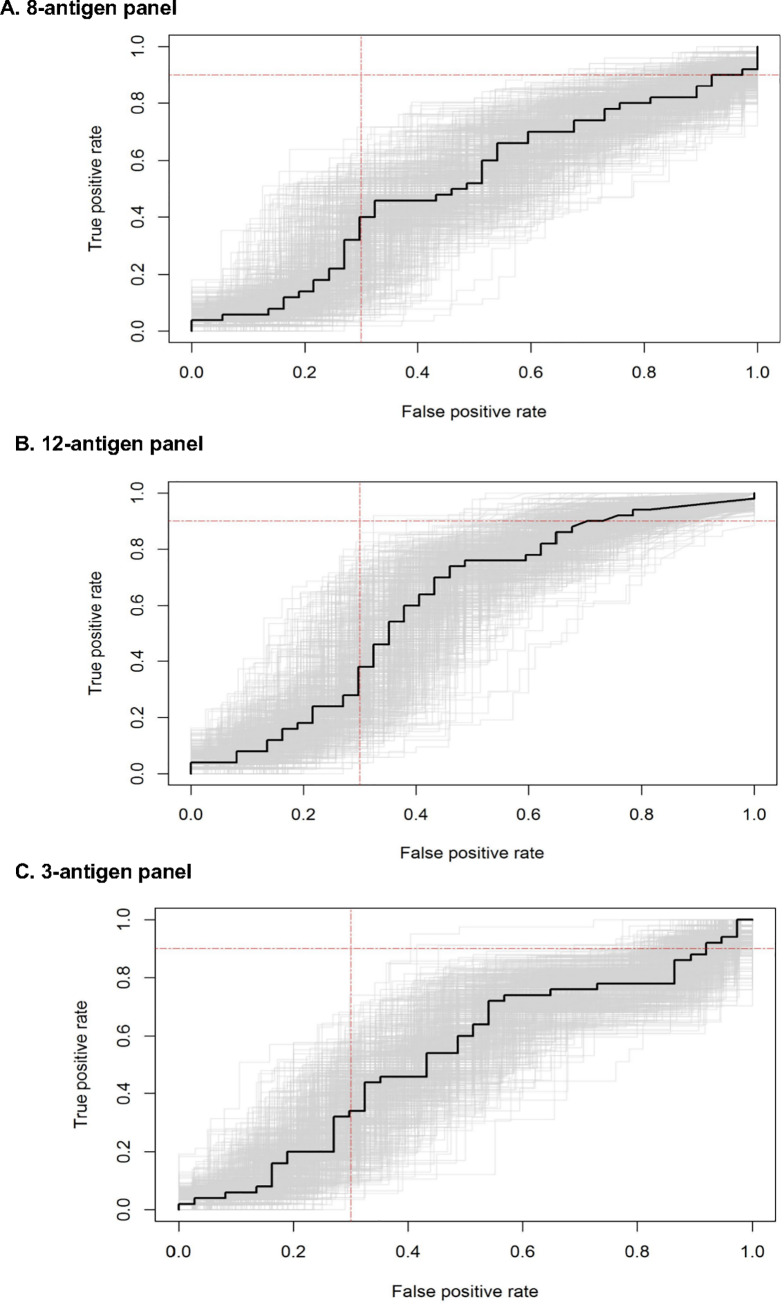
Receiver Operating Characteristic (ROC) curves for TB antigen panels. We generated ROC curves for TB diagnosis among people living with HIV using the following index serological panels: (A) The previously reported 8-antigen panel; (B) The expanded 12-antigen panel; (C) The best performing antigen combination that included Rv0934-P38, Ag85A, and Rv2031-HSPX. The curve in black is the ROC curve on the test set, with 500 bootstrapped ROC curves in grey. The red lines indicate the minimum target performance for a TB triage test with True Positive Rate of 0.9 and False Positive Rate (1- Specificity) of 0.3.

The 8-antigen panel had an AUC of 0.53 (95% CI 0.40–0.66) on the test dataset (Fig 3A). At a set sensitivity threshold of ≥90%, the specificity was 2.2% (95% CI 0–17.7%). If all 12 antigens were used, the AUC was 0.51 (95% CI 0.39–0.64) (Fig 3B), and mean specificity was 8.1% (95% CI 0–23.9%) at a set sensitivity threshold of ≥90%.

### Diagnostic accuracy of best performing antigen combination

**Fig 4:**
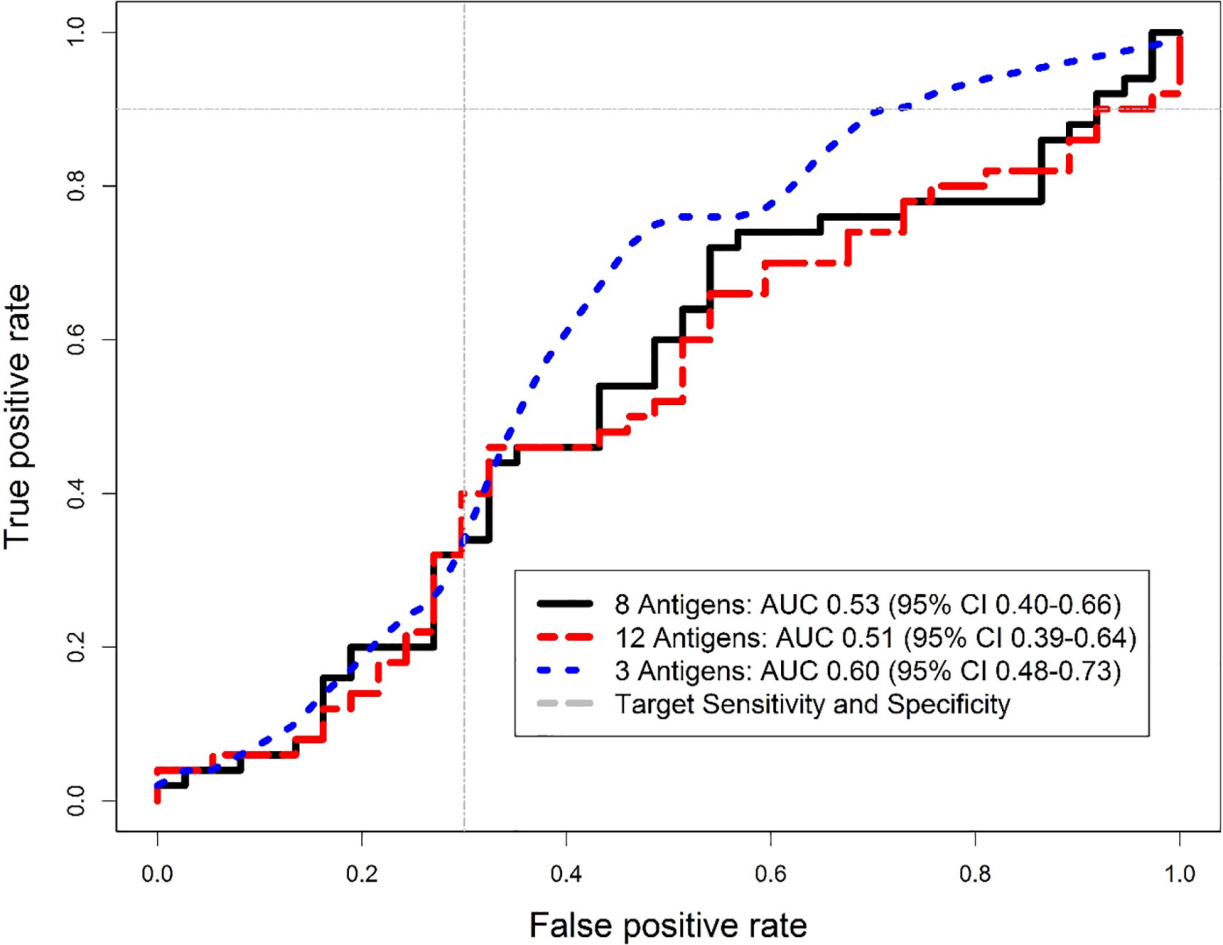
Comparison of antigen panel Receiver Operating Characteristic (ROC) curves. We overlaid the ROC curves of the three multi-antigen serological panels to compare performance and the area under the curve (AUC). The black line is the 8-antigen panel, red is the 12-antigen panel, and blue is the best performing 3-antigen panel (Rv0934-P38, Ag85A, and Rv2031-HSPX). The grey lines indicate the minimum target performance for a TB triage test with True Positive Rate of 0.9 and False Positive Rate (1- Specificity) of 0.3.

After evaluation of every possible antigen combination, a three-antigen signature of Rv0934-P38, Ag85A, and Rv2031-HSPX performed best, with an AUC of 0.64 (95% CI 0.56–0.72) on the training data. When validated on the test set, the AUC was 0.60 (95% CI 0.48–0.73) (Fig 3C), higher than either the 8- or 12-antigen panel (Fig 4).

At a cut-off of 1.61, the sensitivity was 90% (95% CI 78.2–96.7%) and specificity was 29.7% (95% CI 15.9–47%). In comparison, the WHO four-symptom screen was 94% sensitive (95% CI 83.5–98.7%) and 13.5% specific (95% CI 4.54–28.8%). The three-antigen panel was thus 16.2% more specific, but it was not statistically significant (difference 95% CI -4.1 to 36.5%, p = 0.15).

## Discussion

Multi-antigen testing with a microbead immunoassay has previously been shown to achieve the target accuracy thresholds required of a TB triage/screening test [13, 15]. Here, we found that a previously described 8-antigen panel did not achieve the minimum accuracy thresholds for TB screening among a cohort of ART-naïve PLHIV. The addition of four TB-specific antigens did not increase accuracy, and we did not find a combination that could reach the minimum target 90% sensitivity and 70% specificity. However, we identified a novel three-antigen combination of Rv0934-P38, Ag85A, and Rv2031-HSPX that improved specificity, and supports similar methods for further discovery and validation of multi-antigen panels specifically for TB screening among PLHIV.

In examining reasons that the previous panel did not perform as well in this sample, there were major differences between our study and the prior studies in Uganda and Pakistan that identified the TB antigens evaluated in this study [13]. The prior studies included only HIV negative participants, all participants self-presented to hospitals with prolonged cough for at least 2 weeks and 14–45% of participants had smear-negative TB [13, 15]. In contrast, our study occurred at two outpatient HIV clinics where participants were identified through systematic TB screening, 67% of TB cases were smear negative, 40% of participants did not report cough and 9% did not have any TB-related symptoms. Thus, the lower accuracy of the multi-antigen panels may have been due to the earlier stage of disease observed as part of active rather than passive case finding, and consequently smaller differences in antibody response between groups.

In addition, antibody responses in participants were in general low in our study regardless of TB status. The low level antibody responses we observed may be a consequence of the advanced HIV infection in our cohort with median CD4 cell count of 152 cells/μL, which is associated with diminished humoral immune activity [32–34]. While HIV infection is associated with a hypergammaglobulinemia of naïve B cells, it also leads to reduced memory B cells and B cell functional exhaustion that impairs antigen-specific immunity and immunoglobulin diversity [35, 36]. HIV infection is also associated with paucibacillary, smear negative TB disease, where lower levels of circulating antigens and lower titer antibody responses are found [37]. Furthermore, because our sample was a screening cohort in an ambulatory setting, participants may have had a less robust immune response as compared to more severe hospitalized patients. While the antigen panels we examined here did not perform well in the context of TB screening among an outpatient population, additional studies are needed to examine their utility in hospitalized PLHIV with more severe disease.

We identified a three TB antigen panel, consisting of Rv0934-P38, Ag85A, and Rv2031-HSPX that out-performed the previously reported 8-antigen panel as well as all other sets of antigens we examined. Rv0934-P38, also known as PstS1 or 38 kDa antigen, is a phosphate binding protein that has been combined with other antigens for TB diagnosis, though none of these combinations achieved target performance [21, 23, 38, 39]. Ag85A is a secreted protein that is thought to have an important role in *M*. *tuberculosis* pathogenicity [40], and Ag85A peptide sequences have been found to be specific to pulmonary TB [41]. Rv2031-HSPX, also known as alpha-crystallin or 16 kDa antigen, is induced in a hypoxic state, and has been shown to be higher in pulmonary TB compared to household contacts or community controls [39, 42, 43]. We found that all three were significantly higher in TB vs. non-TB participants. This panel likely performed better than the 8-antigen set or all 12 antigens because the unbiased approach was able to include antigens that were significantly different by TB status and could perform best in an independent test set, while excluding antigens that were similar between groups and would worsen diagnostic accuracy. While the three-antigen panel did not meet the WHO TPP for a TB screening test, the specificity of 29.7% was 16.2% higher than the current standard of the WHO four-symptom screen. Although it was not statistically significant, the test set was not powered for this comparison and supports evaluation with a larger sample. Moreover, this is the first report of this particular combination of three antigens, and suggests that a similar unbiased approach may be able to identify novel biomarker combinations for TB screening and diagnosis in PLHIV. These combinations could then be translated into a point-of-care multiplex immunoassay for rapid triage and improved care of this at-risk population.

As fewer antigens are thought to induce an antibody response in HIV-infected individuals with TB [24, 44], high throughput antigen screening methods could be promising to identify novel serological biomarkers in HIV-TB co-infection [24, 45]. For example, a high-density nucleic acid programmable protein array was used to evaluate the sera of HIV infected and uninfected individuals in the United States and South Africa, and identified four new antigens [46]. When coupled with four known TB antigens, the AUC of this antigen set for HIV positive individuals in South Africa was 0.723. Although the specificity of this antigen set was 50% at a sensitivity threshold of 80%, these results provide initial evidence that high-throughput approaches may be useful to identify antigens useful for TB screening among PLHIV. Serologic responses to TB-specific antigens could also be added with host markers to improve specificity and achieve the target threshold for a triage test; for example, a study in HIV negative individuals added Ag85B to four host proteins and achieved 86% sensitivity and 69% specificity [47].

We were able to examine the utility of serologic TB screening in HIV with previously validated antigens, by taking advantage of a well-characterized, prospective cohort study of ART-naïve PLHIV with complete microbiological investigation for TB and a high throughput, multiplex immunoassay. Our study does have some limitations. The observed small differences in antibody responses between participants with and without TB could have been caused by false negativity of culture results in smear negative disease, or degradation of the frozen plasma specimens. However, Xpert MTB/RIF results correlated with culture results, samples did not have a prior freeze-thaw cycle and were continuously stored at -80°C with standard quality control protocols, and were tested within a year of the end of enrollment, making these explanations unlikely. Because of the low CD4 cell count distribution, we could not stratify by CD4 category to determine if higher CD4 cell count increased antibody response and performance, but those with low CD4 cell count are at highest risk of TB disease.

Despite the growing body of literature on TB biomarkers, a non-sputum-based triage test at the point of care remains elusive. Key obstacles include the limited validation and translation of promising biomarkers to relevant populations [9]. Our study sought to address this barrier by applying a previously used multiplex microbead immunoassay for TB screening among PLHIV. While the antigen panels we tested did not reach the target accuracy thresholds in our sample of outpatient PLHIV being screened for TB, they may still be useful in other groups including HIV negative or hospitalized individuals, and need further validation. Our work also highlights the need to discover and validate multi-antigen panels specifically for TB screening among PLHIV.

## Supporting information

S1 File Data of antibody responses to TB antigens among people living with HIV, Kampala, Uganda. Values are the log median fluorescence intensity (MFI) minus the Bovine Serum Albumin (BSA) response. Zero represents an untransformed MFI value of 0. “tbpos” is defined as having TB if 1, and without TB as 0.
(CSV)

S1 Table Comparison of median antibody responses to *M*. *tuberculosis* antigens by pulmonary TB status in Kampala, Uganda. IQR: interquartile range; MFI: Median Fluorescence Intensity; TB: tuberculosis. a. Calculated by permutation testing; b. Log MFI 0 values represent an untransformed MFI of 0.
(PDF)
