# Supplementary material for: Evaluation of multi-antigen serological screening for active tuberculosis among people living with HIV
Source: PLoS One. 2020 Jun 4;15(6):e0234130. doi: 10.1371/journal.pone.0234130 (PMC7272080; doi:10.1371/journal.pone.0234130)
Supplement: S1 Table — IQR: interquartile range; MFI: Median Fluorescence Intensity; TB: tuberculosis. a. Calculated by permutation testing; b. Log MFI 0 values represent an untransformed MFI of 0. (PDF) [file pone.0234130.s002.pdf]

S1 Table. Comparison of Median Antibody Responses to *M. tuberculosis* antigens by pulmonary TB status in Kampala, Uganda

TB antigen	Log MFI without TB Median (IQR)[b]	Log MFI with TB Median (IQR)[b]	p-value[a]
Rv3881	2.41 (0-4.15)	2.88 (0.10-4.25)	0.09
Rv0934-P38	0 (0-0)	0 (0-0.89)	0.02
Ag85A	2.35 (1.38-3.06)	2.78 (1.8-3.26)	0.01
Ag85B	2.30 (0.66-3.22)	2.48 (0.53-3.73)	0.3
Ag85C	2.88 (1.25-3.81)	3.39 (2.26-3.93)	0.02
Rv3873	4.13 (3.36-5.02)	4.31 (3.77-5.00)	0.05
Rv3841-BfrB	2.85 (1.78-4.02)	3.27 (2.15-4.15)	0.06
Rv2878c-MPT53	2.44 (0-4.53)	2.11 (0-4.33)	0.77
Rv2031-HSPX	0 (0-0.51)	0 (0-2.57)	0.01
ESAT-6	0 (0-1.98)	0 (0-3.54)	0.04
CFP-10	0 (0-2.23)	0 (0-2.80)	0.21
Rv1980-MPT64	4.56 (3.68-5.57)	4.74 (4.16-5.65)	0.04

IQR: interquartile range; MFI: Median Fluorescence Intensity; TB: tuberculosis

a. Calculated by permutation testing
b. Log MFI 0 values represent an untransformed MFI of 0
